# Spatiotemporal impacts of human activities and socio-demographics during the COVID-19 outbreak in the US

**DOI:** 10.1186/s12889-022-13793-7

**Published:** 2022-08-01

**Authors:** Lu Ling, Xinwu Qian, Shuocheng Guo, Satish V. Ukkusuri

**Affiliations:** 1 Lyles School of Civil Engineering, Purdue University, West Lafayette, USA; 2 Department of Civil, Construction and Environmental Engineering, The University of Alabama, Tuscaloosa, USA

**Keywords:** Disease propagation, Human activity, Social-demographic characteristics, Spatial and temporal heterogeneity, Geographically and temporally weighted regression

## Abstract

**Background:**

Understanding non-epidemiological factors is essential for the surveillance and prevention of infectious diseases, and the factors are likely to vary spatially and temporally as the disease progresses. However, the impacts of these influencing factors were primarily assumed to be stationary over time and space in the existing literature. The spatiotemporal impacts of mobility-related and social-demographic factors on disease dynamics remain to be explored.

**Methods:**

Taking daily cases data during the coronavirus disease 2019 (COVID-19) outbreak in the US as a case study, we develop a mobility-augmented geographically and temporally weighted regression (M-GTWR) model to quantify the spatiotemporal impacts of social-demographic factors and human activities on the COVID-19 dynamics. Different from the base GTWR model, the proposed M-GTWR model incorporates a mobility-adjusted distance weight matrix where travel mobility is used in addition to the spatial adjacency to capture the correlations among local observations.

**Results:**

The results reveal that the impacts of social-demographic and human activity variables present significant spatiotemporal heterogeneity. In particular, a 1% increase in population density may lead to 0.63% more daily cases, and a 1% increase in the mean commuting time may result in 0.22% increases in daily cases. Although increased human activities will, in general, intensify the disease outbreak, we report that the effects of grocery and pharmacy-related activities are insignificant in areas with high population density. And activities at the workplace and public transit are found to either increase or decrease the number of cases, depending on particular locations.

**Conclusions:**

Through a mobility-augmented spatiotemporal modeling approach, we could quantify the time and space varying impacts of non-epidemiological factors on COVID-19 cases. The results suggest that the effects of population density, socio-demographic attributes, and travel-related attributes will differ significantly depending on the time of the pandemic and the underlying location. Moreover, policy restrictions on human contact are not universally effective in preventing the spread of diseases.

## Background

As of June 2022, the severe acute respiratory syndrome coronavirus 2 (SARS-CoV-2), the aetiological agent of coronavirus disease 2019 (COVID-19), has infected 84 million people and caused more than 1 million deaths [1] in the US. The main routes of SARS-CoV-2 entry and transmission are “contact”, “droplet”, and “airborne” [2]. In light of the severe consequences from the COVID-19 outbreak, different public authorities quickly responded to the outbreak through various strategies, including the declaration of emergency, travel restrictions, city lock-down, and enforcing social distancing [3, 4]. If properly followed and executed, these measures serve as the crucial first steps to limit physical contact and mitigate the extent of the outbreak before a vaccine is available. Nevertheless, under similar mitigation measures, significant differences are observed in the number of reported infections and the mortality rate across the US [5]. This motivates us to explore the underlying factors that result in the heterogeneous disease dynamics for assisting the disease mitigation policies in the remaining phase of the COVID-19 and better preparing against future risks of unknown infectious diseases.

As mentioned in the WHO study for the 2009 H1N1 pandemic [6], in addition to the pathological variables, the extent of the disease outbreak may be attributed to various non-epidemiological factors, including mobility level, social-demographics, pre-existing conditions of the population [7], quality of health services, travel patterns, social network [8–11], ecological factors [6, 12], etc. But our knowledge of the precise impacts of these factors is very limited, primarily due to the lack of data that may enable the nexus between disease dynamics and the possible contributing factors. With recent advances in ubiquitous computing and epidemiology and the wide adoption of smartphones in the past decade, we are now able to monitor human activities at a fine spatiotemporal level and overlay such dynamics with high-resolution trajectories of disease outbreaks. This, together with the available data on socioeconomic, demographics, and historical daily commuting patterns, provides an unprecedented opportunity to scrutinize the impacts of non-epidemiological factors and comprehensively evaluate how these factors drive the fate of the disease outbreak across the US.

Existing studies have related social-demographic characteristics and human activity with the spread of the COVID-19. The social-demographic structure of the population is demonstrated to have a significant effect on the fatality rate. An early study in China [13] suggested that people with an age greater than 80 years older have the highest fatality rate of 14.8%, and similar findings were obtained from studies in other countries [14, 15]. In addition, studies [16, 17] revealed the existence of racial disparities among the Whites, the blacks, the Asians, and the Hispanics in the COVID-19 outbreak. In particular, nearly 20% of the US counties had a disproportionate black population [18], and they accounted for 52% of the confirmed cases and 58% of the deaths nationally. Except for demographic factors, the social and economic factors are also found to affect the fate of the COVID-19 outbreak. The study [19] suggested that households with the lowest income level are six times less likely to be able to work from home and three times less likely to be able to self-isolate in the UK during the COVID-19. Besides, Stojkoski et al. [20] mentioned that the high-income population is more resilient to being infected by the COVID-19. Finally, extensive efforts have shown that human activities and mobility dynamics are dominating factors that facilitate the spread of infectious diseases [21–24]. Studies suggested that information propagation and commercial activity patterns co-affect the epidemic propagation [25–27]. Nevertheless, Lima et al. [28] provided evidence that restricting mobility may not eliminate the diseases. And Bajardi et al. [29] recommended that stricter regimes of travel reduction would have led to a delayed outbreak of two weeks based on the study of the 2009 H1N1 pandemic.

The aforementioned studies highlighted the significant roles played by mobility-related and social-demographic factors in the disease spreading process. Nevertheless, few studies examined the collective impacts of non-epidemiological factors on the spatiotemporal dynamics of infectious disease. In addition, the impacts of these influencing factors were primarily assumed to be stationary over time and space in the existing literature. The lack of consideration of these aspects will fail to reveal the interdependencies among modeling determinants and may result in biased model estimations.

To address the issues, the study aims to introduce a quantitative approach, named mobility-augmented geographically and temporally weighted regression model (M-GTWR), to investigate the heterogeneous effects of non-epidemiological factors on the spreading dynamics of the COVID-19. By relating pre-pandemic inter-county traffic data with the spatial adjacency, the M-GTWR quantifies the spatiotemporal effects of the social-demographic characteristics and human activity on the weekly average daily confirmed cases in the US. Our results suggest that counties with a high percentage of black population, a high household income level, a low education level, and a high unemployment rate are associated with more weekly average daily confirmed cases. Moreover, the impact of human activity is found to differ spatially. Grocery and pharmacy activities only show positive and statistically significant effects on the COVID-19 cases in rural counties, and the effects of the public transit activities are tightly related to the work from home policy and reopening strategies.

## Methods

### Study area

**Fig. 1:**
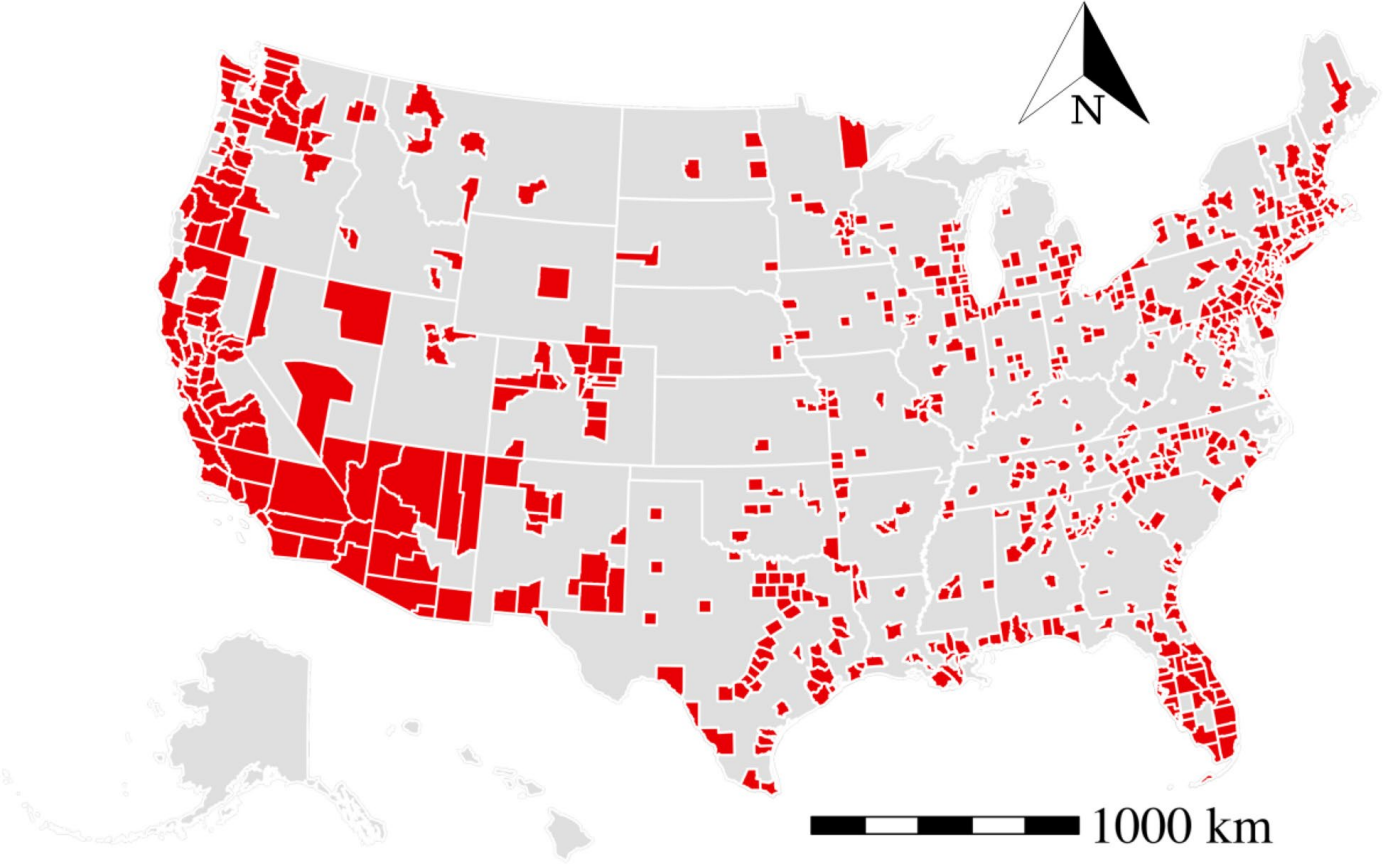
The spatial distribution of selected counties (in red) in the US

We investigate the COVID-19 dynamics in the US counties. There are 3141 counties in the US, and the counties present a significant variation of reported daily cases. To ensure that the disease dynamics are statistically meaningful, we target the counties with at least 100 confirmed cases from March 23, 2020 to December 13, 2020. In addition, counties with incomplete data are also removed. Finally, we keep the counties within 48 contiguous states. The preprocessing results in 699 selected counties that cover both metropolitan areas (592) and non-metropolitan areas (107) according to the definition in the rural-urban commuting area code [30]. And the spatial distribution of the study areas (red-colored counties) is shown in Fig. 1. The selected counties cover $79\%$ of the total US population. We report that the processed data provide a reasonable scale to understand the non-epidemiological determinants of the COVID-19 propagation and link with the underlying effects of the social-demographics characteristics and human activities on disease propagation in the metropolitan counties in the US.

### Data Description

#### Dependent variables

**Fig. 2:**
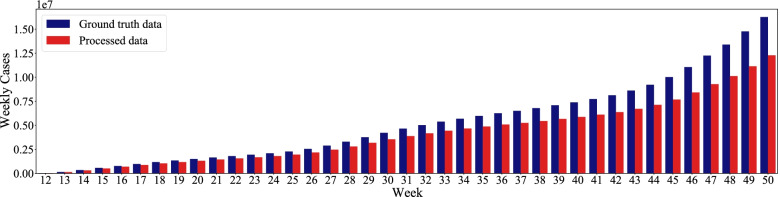
The number of weekly average daily confirmed cases between processed counties and all counties in US

The dependent variables used in the study are the number of weekly average daily confirmed cases at the county level, which are obtained from the Center for Systems Science and Engineering at Johns Hopkins University [5]. We use the weekly average daily confirmed cases rather than daily cases to smooth the daily fluctuations in the data. As suggested in other studies [13], there is a time delay between the date when an individual was actually infected and the reported date, which is usually two weeks for the COVID-19. In addition, human activity dynamics were reflective of the reporting date. To ensure the consistency between the disease and mobility dynamics, we apply a two-week delay to the human activity data to match the disease data. We report that the number of confirmed cases in the selected counties accounted for 75.5% of the total cases during the study period in the US, as shown in Fig. 2. These indicate that the dependent variables in the selected counties are representative of the general disease dynamics in the metropolitan areas in the US.

#### Explanatory variables

The explanatory variables used in our study contain both the dynamic variables that changed over time as the disease progressed and the static variables that remained constant during the study period. Table 1 summarizes the selected variables and their summary statistics.

**Table 1:** Definition and descriptive statistics of explanatory variables

Variables	Definitions	Mean	S.D.	VIF
Demographic				
POPD	Population density (per square km)	1210.83	4374.67	2.53
OLDP	The percentage of older population (%)	15.56	4.40	1.65
BLAP	The percentage of black population or African American population (%)	12.09	12.95	2.29
ASIP	The percentage of Asian population (%)	3.89	4.48	2.14
LATP	The percentage of Hispanic Latino population (%)	15.53	16.31	2.07
Socioeconomic				
BDHP	Percentage of population having at least bachelor’s degree (%)	31.26	10.71	7.11
MHIC	Yearly mean household income ($)	82836	21101	6.68
CLUE	Citizen labor force unemployment rate (%)	5.91	1.81	2.45
Travel				
MILE	Public road mileage (meter)	5238.21	5128.13	1.32
WFHP	Percentage of population work at home (%)	4.83	1.88	2.21
PTAP	Percentage of population taking public transit (%)	3.09	6.86	2.09
MTAT	Mean commuting time (minutes)	24.94	5.01	2.60
Human Activity				
GPPC	Change of grocery and pharmacy activity (%)	-3.03	10.80	3.27
WOPC	Change of workplace activity (%)	-27.70	14.49	4.74
TSPC	Change of transit stations activity (%)	-19.06	23.27	2.24

##### Dynamic variables

**Fig. 3:**
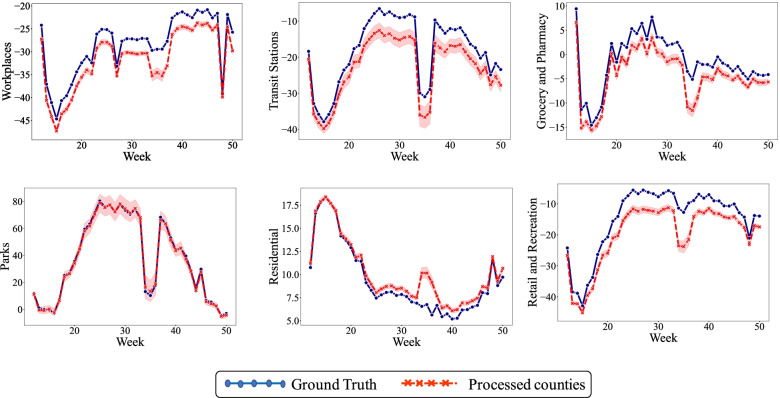
The dynamic variables of the processed county and all counties in US

The dynamic variables in the study are obtained from the Google Mobility Report [31]. The Google Mobility Report describes the change of daily activities in terms of recreation activity (RRPC), park activity (PAPC), residential activity (RAPC), grocery and pharmacy activity (GPPC), transit activity (TSPC), and workplace activity (WOPC) from the baseline value. The baseline value is the median value for the corresponding day of the week between January 3, 2020 and February 6, 2020. The dataset demonstrates the changes in visiting frequency of a particular activity category at individual counties and is also indicative of the activity intensity on the corresponding day of the week. To be consistent with the dependent variables, we calculated the average daily activity in a week. Then, we visualize the mean and standard deviation of the dynamic variables in the selected counties and their comparison with all counties in US in Fig. 3. Similar to the dependent variables, we observe that the dynamic variables in selected counties also resemble the trends of the entire population in US.

To eliminate the estimated bias from the association effects and multicollinearity issue, we tested the Pearson correlation and variance inflation factor(VIF) among dynamic variables in Table 2. It is observed that the Pearson correlations of the GPPC, TSPC, and WOPC are less than 0.6, and the VIF values are less than 10. However, the Pearson correlations among the RRPC, PAPC, and RAPC are near 0.7, which indicates a high correlation among these variables. Therefore, we selected GPPC, TSPC, and WOPC as model input.

**Table 2:** Pearson product-moment correlation coefficient for explanatory variables

	POPD	OLDP	BLAP	ASIP	LATP	MILE	BDHP	MHIC	CLUE	WFHP	PTAP	MTAT	GPPC	WOPC	TSPC
POPD	1														
OLDP	-0.08	1													
BLAP	0.1	-0.19	1												
ASIP	0.31	-0.25	-0.05	1											
LATP	0.06	-0.2	-0.22	0.13	1										
MILE	-0.04	-0.06	-0.13	0.13	0.33	1									
BDHP	0.26	-0.16	-0.06	0.43	-0.21	-0.08	1								
MHIC	0.26	-0.12	-0.21	0.49	-0.02	-0.02	0.49	1							
CLUE	0.01	0.02	0.37	-0.13	0.26	0.23	-0.46	-0.47	1						
WFHP	0.05	0.21	-0.28	0.2	-0.01	0.11	0.47	0.48	-0.29	1					
PTAP	0.46	-0.13	0.08	0.49	0.08	-0.01	0.45	0.47	-0.02	0.1	1				
MTAT	0.23	-0.06	0.04	0.37	0.1	0.08	0.22	0.45	-0.01	0.24	0.41	1			
GPPC	-0.19	-0.06	0.08	-0.25	-0.1	0.01	-0.36	-0.33	0.12	-0.25	-0.28	-0.19	1		
WOPC	-0.29	0.17	0.07	-0.49	0.04	0.01	-0.52	-0.57	0.23	-0.29	-0.48	-0.32	0.47	1	
TSPC	-0.27	0.06	-0.02	-0.4	-0.09	-0.07	-0.45	-0.42	0.09	-0.24	-0.46	-0.26	0.49	0.44	1

##### Static variables

In this study, the static variables include the county level demographic factors, socioeconomic factors, and travel-related information. The demographic factors include the total population, older population, white population, black or African American, Asian population, Hispanic Latino population, and land area. They are collected from the US Census Bureau’s MAF/TIGER Geodatabases [32] and the 2016 American Community Survey (ACS) [33]. In light of the varying size of the selected counties, we calculated the population density (POPD) by dividing the total population by the land area. Besides, we measured the percentage of race type by dividing the population in each race category by the total population. The socioeconomic factors in our study include the percentage of the population having at least a bachelor’s degree (BDHP), yearly mean household income (MHIC), and the citizen labor force unemployment rate (CLUE). They are collected from the 2016 ACS [33]. The socioeconomic factors serve as indirect measures to probe how people may respond to the preventative measures and the economic resilience of the community against disease outbreaks.

In addition to the demographic and socioeconomic variables, we also include several travel-related factors as human activity intensity measures before the COVID-19. The travel-related factors include travel mode factors and public road mileage (MILE). The travel mode factors in our study contain the percentage of the population working at home (WFHP), the percentage of the population taking public transit (PTAP), and the mean commuting time (MTAT). These variables are obtained from the 5-Year ACS statistic (ACS 2011 to 2015) [34]. Except for the above variables, public road mileage is another crucial travel-related factor to reflect the intensity of economic activities in the county. The public road is described as any road under the jurisdiction maintained by a public authority. We collected the public road mileage from the 2018 Public Road Geodatabase [35]. Table 2 presents the Pearson correlation coefficients of the static factors, where most of the Pearson correlation coefficients of the static variables are less than 0.4. Some variables having correlations below 0.6 are also included in our model because they capture significant variations and provide non-overlapping effects for the dependent variables (we take the public road mileage as an explanatory factor instead of the public road mileage density to avoid the high correlation between public road mileage density and population density).

#### Traffic flow data

The traffic flow data are used to cooperate with the spatial adjacency and serve as a kernel function in the M-GTWR model to describe the spatial structure among counties. The traffic flow data include the road commuting flow and airline flow, which are gathered to supplement the spatial distance with the real travel connections among counties during the COVID-19 outbreak. The distance data are directly obtained from the shapefile provided by the US Department of Transportation and Bureau [36]. The road commuting flow among counties is collected from the average 5-Year ACS statistic at the county level [34] and describes the traffic connections between residence counties and workplace counties. And the geographical airline passenger flow among airports is provided by the US Department of Transportation and Bureau. The Bureau of Transportation Statistics offers quarterly airline and airport origination and destination survey (DB1B) within the US, which is a 10% sample of airline tickets (passengers) from reporting carriers. The DB1B has records of airline passenger volumes in US airports, but it does not contain information of airline flow among counties. To obtain the airline flow, we apply the airline traffic flow assignment method based on the origination and destination airport passenger volumes from DB1B.

### Mobility-augmented geographically and temporally weighted regression model

In light of the heterogeneous disease dynamics in the US, conventional global regression techniques are no longer appropriate by assuming that all determinants are stationary over space and time. The geographically and temporally weighted regression model (GTWR) [37] is an effective method to account for the spatial and temporal nonstationarity issues and provides more interpretable estimations for the influencing factors during the COVID-19 pandemic. Considering that we have a series of *N* observations $(Y_{1},X_{1}),(Y_{2},X_{2}),...,(Y_{N},X_{N})$ over time and space, each at location $(u_{i},v_{i})$ and at time $t_{i}$, the GTWR model can be formulated as follows:

1 $$\begin{aligned} Y_{i} = \beta _{0} (u_{i}, v_{i}, t_{i}) + \sum \limits _{k=1}^{n} \beta _{k} (u_{i}, v_{i}, t_{i}) X_{ik} + \epsilon _{i} \end{aligned}$$

where $Y_{i}$ and $X_{ik}$ refer to the dependent variable and the *k*th explanatory variable of the *i*th observation. $\epsilon _{i}$ is the error term with $\epsilon _{i} \sim \mathcal {N}(0,\sigma ^{2})$. $\beta _{0} (u_{i}, v_{i}, t_{i})$ is the intercept, and $\beta _{k} (u_{i}, v_{i}, t_{i})$ is the regression parameter obtained as:

2 $$\begin{aligned} \hat{\beta }(u_{i}, v_{i}, t_{i}) = [X^{T} W(u_{i}, v_{i}, t_{i}) X ]^{-1} X^{T} W(u_{i}, v_{i}, t_{i}) Y \end{aligned}$$

where $W(u_{i}, v_{i}, t_{i})$ is the spatiotemporal weight matrix.

In the GTWR model, the weight between two observations is estimated solely based on the spatial distance and the time gap. Nevertheless, the disease propagation shall not only be influenced by geographic distance but mobility connections among locations. Therefore, we developed a M-GTWR model that incorporates the great circle distance-based weight matrix with the mobility-based weight (the components include airline volume and commuting volume) to improve the baseline GTWR model [37, 38]. The standardized form of each component is proposed in this study to reduce the fluctuation of the components in the mobility-augmented weight function $d^{M}_{ij}$ between node *i* and *j*.

3 $$\begin{aligned} d^{M}_{ij} = \frac{d_{ij}^{S}}{\sigma \left( {d_{ij}^{S}}\right) } + \tau _{air}\exp \left[ - \frac{N^{air}_{ij}}{\sigma \left( N^{air}_{ij} \right) } \right] + \tau _{commuting}\exp \left[ - \frac{N^{commuting}_{ij}}{\sigma \left( N^{commuting}_{ij} \right) } \right] \end{aligned}$$

where $\tau _{air}$ and $\tau _{commuting}$ represent the parameters for the standardized airline volume and the standardized commuting volume; $N_{ij}^{air}$ and $N_{ij}^{commuting}$ denote the real airline volumes and real commuting volumes; $\sigma \left( {d_{ij}^{S}}\right)$, $\sigma \left( N^{air}_{ij} \right)$, and $\sigma \left( N^{commuting}_{ij} \right)$ are the prior bandwidth to standardize each component in the mobility-augmented weight matrix.

Besides, the function of the mobility-augmented weight matrix combining the temporal distance matrix ($d_{ij}^{T}$) is shown below. Note that the geographically weighted regression (GWR) model with mobility-augmented weight matrix (M-GWR) can be achieved when $\lambda =1$. Besides, we apply the Gaussian kernels into the M-GTWR model.

4 $$\begin{aligned} d_{ij}^{MST} = \lambda d_{ij}^{M} + (1- \lambda ) d_{ij}^{T} +2 \sqrt{\lambda (1- \lambda ) d_{ij} ^{M} d_{ij} ^{T}} \end{aligned}$$

During the model calibration, we first apply the cross-validation (CV) method proposed by Shao [39] to select the optimal bandwidth $h^{MST}$ for M-GTWR [40], choose the spatiotemporal effect parameter $\lambda$ [37], and calibrate parameters ($\tau _{air}, \tau _{commuting}$). Then, the corrected Akaike Information Criterion (AICc) is used to calibrate the trade-off between goodness of fit and degrees of freedom [40]. Finally, we verify the effectiveness of the M-GTWR model based on the analysis of variance (ANOVA) method.

### Airline traffic assignment

Since the airline passenger volume has potential seasonal fluctuation, we used the DB1B market records of the first quarter of 2019 to infer the airline travel connections in the first quarter of 2020 (when COVID-19 started) in this study. The dataset contains 420 original airports and 419 destination airports, which serve 396 original cities and 394 destination cities in the US. To obtain the airline flow among the selected counties, we first assign the number of passengers from the origin airport to the nearby counties that are within the radiation range using the below distance-based gravity model [41]:

5 $$\begin{aligned} w_{ij}=C\times {\frac{(P_{i})^{\alpha }\times (P_{j})^{\gamma }}{f(d_{ij})}} \end{aligned}$$

In the equation, *C* is a proportionality constant, $\alpha$ and $\gamma$ tune the dependence related to the population size of each county. The distributed weight $w_{ij}$ is positively related to the product of the population of served county $P_{i}$ and the population of the airport located county $P_{j}$, and negatively related to the distance $d_{ij}$ between the two counties. And $f(d_{ij})$ is a distance-dependent functional form, which assumes to be an exponential law for the dis-attraction between two counties and is defined as:

6 $$\begin{aligned} f(d_{ij})=e^{(\beta d_{ij})} \end{aligned}$$

According to [41], the empirical PDF of the connected airline volume reaches the summit when the distance between the two areas is around 250km and decays exponentially afterward. The parameters $\alpha$, $\gamma$, and $\beta$ used in the study are, therefore, obtained from their statistical analysis: $\alpha$ is 0.46, $\gamma$ is 0.64, and $\beta$ is 0.0122 when the distance is less than or equal to 300*km*, and $\alpha$ is 0.35, and $\gamma$ is 0.75 when the distance is great than 300*km*.

The assigned air travel demand $PD_{i}$ of county *i* is $PD_{i}=TD_{j} w_{ij}$, where $TD_{j}$ is the total passengers volume of the original airport *j*. After distributing the passengers demand of served county in the US, we then conduct a similar approach to get the assignment weight $w^{'}_{i^{'}j}$ between the destination airport $i^{'}$ and the county *j*. Finally, the airline passengers’ demand from county *i* to county *j* is calculated as

7 $$\begin{aligned} PD_{ij}=PD_{i}\times w^{'}_{i^{'}j} \end{aligned}$$

### Model calibration

#### Spatial autocorrelation and heterogeneity test

To understand the effects of influencing factors on the COVID-19 propagation, we first assess whether there are significant spatial nonstationarity and autocorrelation of the dependent variables over the study period. In this study, we apply the Breusch-Pagan(BP) test to examine the spatial heterogeneity of the weekly average daily confirmed cases. The BP test is a classic approach to detect spatial heterogeneity [42]. The null hypothesis in the BP test is that the error variables are equal in all areas: $\sigma ^{2}_{1}=\sigma ^{2}_{2}=\sigma ^{2}_{i}=\dots =\sigma ^{2}$. The alternative hypothesis is that there should be at least one location *i*, such that $\sigma ^{2}_{i} \ne \sigma ^{2}$. Besides, we use the adjusted Moran’s I test to examine the spatial autocorrelation of the weekly average daily confirmed cases that varies from time, which is proposed by Gao et al. [43]. Based on the tested BP value being 332.77(***), we reject the null hypothesis of homoscedasticity for weekly average daily confirmed cases in the 38 weeks. In addition, adjusted Moran’s I is 0.573(***) with Z scores being 445.11. The result demonstrates the spatial misspecification of weekly average daily confirmed cases. Moreover, since the Z-score values are positive in the number of weekly average daily confirmed cases, it implies that the spatial distribution of counties having weekly average daily confirmed cases is more likely to be spatially clustered.

#### Mobility-augmented weight matrix calibration

**Fig. 4:**
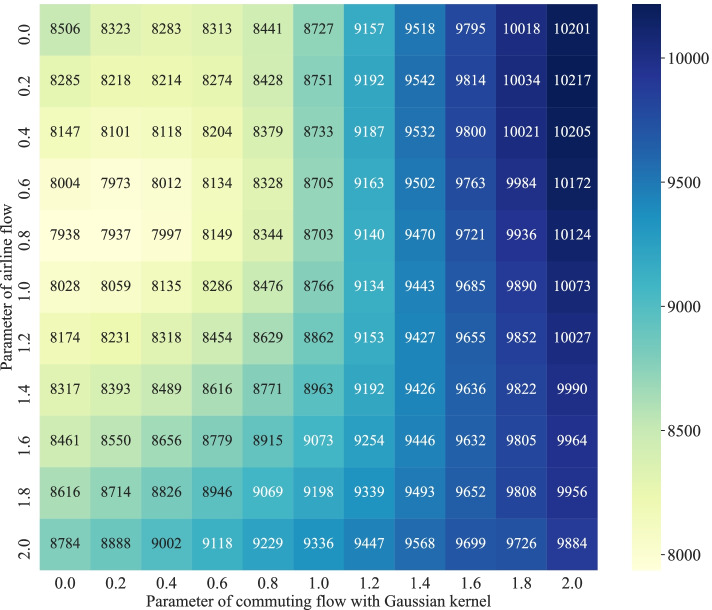
The parameter calibration in the M-GTWR model

The mobility-augmented weight matrix in the M-GTWR model is incorporated by the standardized form of the great circle distance with the mobility connection (airline flow and commuting flow). To calibrate the parameters($\tau _{air}, \tau _{commuting}$), we conducted the CV to verify the performances of different parameter combinations. The results are shown in Fig. 4. Based on the analysis, the optimal parameters for the combination of the standardized mobility connection is 0.8 for the airline flow and 0.2 for the commuting flow, with the corresponding AICc being 7937 in the M-GTWR model. And the optimal $\lambda$ is 0.98, which means the spatial effect is the dominant effect in the spatiotemporal relationship. The final weight matrix in the M-GTWR model is:

8 $$\begin{aligned} d^{M} _{ij} = \frac{d_{ij}^{S}}{\sigma \left( {d_{ij}^{S}}\right) } + 0.8\times \exp \left[ - \frac{N^{air}_{ij}}{\sigma \left( N^{air}_{ij} \right) } \right] + 0.2\times \exp \left[ - \frac{N^{commuting}_{ij}}{\sigma \left( N^{commuting}_{ij} \right) } \right] \end{aligned}$$

#### Model comparisons

After finalizing the modeling parameters, we then evaluate whether the proposed M-GTWR model is superior to other benchmarks in characterizing the spatial and temporal variations of the weekly average daily confirmed cases and offering better explanatory power for the COVID-19 case. The selected benchmarks include the base GTWR model, the M-GWR and GWR model that only consider spatial heterogeneity, and the (ordinary least squares) OLS model that assumes stationarity. We use the ANOVA to compare the improvements in the residual reduction among the candidate models. This approach is also adopted by [37] for model comparisons in studying the spatiotemporal variations of real estate prices. The results of the ANOVA test are summarized in Table 3.

**Table 3:** ANOVA comparison between GWR and OLS models

Source of variance	RSS	DF	MS	F-test	*P*-value
OLS residuals	3205	15	2136.7		
GWR-basic residual	18988	15856	1.2	1784	***
M-GTWR residual	18484	15949	1.2	1844	***
GTWR-basic residual	6168	1456	4.2	504	***
M-GTWR residual	2869	12025	0.2	8956	***
GWR-basic/OLS improvement	13063	1423	9.2		
M-GTWR/OLS improvement	13567	1329	10.2		
GTWR-basic/OLS improvement	25883	1306	19.8		
M-GTWR/OLS improvement	29182	5253	5.6		
M-GTWR/GWR-basic improvement	16119	3830	4.2		
M-GTWR/M-GWR improvement	15615	3924	4.0		
M-GTWR/GTWR-basic improvement	3299	3947	0.8		

The statistics demonstrate the significance of the spatial and temporal nonstationarity of the weekly average daily confirmed cases in the study area over the period. And it is preferable to adopt the GWR-based model instead of the OLS model. Besides, the comparison between the GWR model and the GTWR model asserts the importance of considering the temporal nonstationarity of the data. Finally, we also observe that the mobility-augmented weighting scheme achieves notable improvements in modeling residual for the GWR model and the GTWR model and that the improvements are statistically significant. Therefore, the results support the superiority of the M-GTWR model over all other alternatives in representing COVID-19 dynamics in the US.

## Results

The estimated results of the M-GTWR model are obtained in Table 4. The M-GTWR model addresses the nonstationarity issue for the fundamentally heterogeneous and dynamic disease propagation and provides more efficient estimates by assuming the effects of the influencing factors are spatiotemporal heterogeneous. Compared with the global OLS model, the M-GTWR model improves AICc and adjusted $R^{2}$ from 59779.59 and 0.54 to 26309.13 and 0.94.

**Table 4:** Estimates of the M-GTWR model

Variables	Min	Max	Median	Lower quartile	Upper quartile
Intercept	-73.19	16.60	-13.81	-28.58	-4.94
Demographic					
Log.POPD	0.18	1.20	0.63	0.57	0.69
OLDP	-13.13	8.96	-1.30	-4.05	0.85
BLAP	-0.05	0.05	0.02	0.01	0.02
ASIP	-0.15	0.12	-0.03	-0.06	-0.01
LATP	0.02	0.05	0.01	0.01	0.02
Socioeconomic					
CLUE	-0.40	0.35	-0.05	-0.09	0.01
BDHP	-0.17	0.07	-0.01	-0.02	0.00
Log.MHIC	-1.90	6.71	0.83	0.13	1.86
Travel					
Log.MILE	0.44	1.82	1.03	0.91	1.13
WFHP	-0.38	0.27	-0.07	-0.13	-0.03
PTAP	-0.08	0.17	0.01	-0.01	0.02
Log.MTAT	-3.20	3.69	0.22	-0.44	0.88
Human Activity					
GPPC	-0.06	0.05	-0.01	-0.02	0.01
WOPC	-0.11	0.08	-0.01	-0.03	0.01
TSPC	-0.04	0.02	-0.01	-0.01	0.01
AIC			21356.98		
AICc			26309.13		
$R^{2}$			0.96		
Adjusted $R^{2}$			0.94		

### The impacts of the demographic variables

#### Population density (POPD)

Instead of the static effects of the influencing factors estimated in the global OLS model, our findings show the effects of the factors vary among regions and time. For estimates of the demographic variables, the coefficient of population density is positive to weekly average daily confirmed cases, and the estimates are statistically significant in the studied counties from the 12th week and 48th week. In particular, the median elasticity of the population density shows that a 1% increase in the population density leads to a 0.63% increase in the weekly average daily confirmed cases.

#### Percentage of older population (OLDP)

**Fig. 5:**
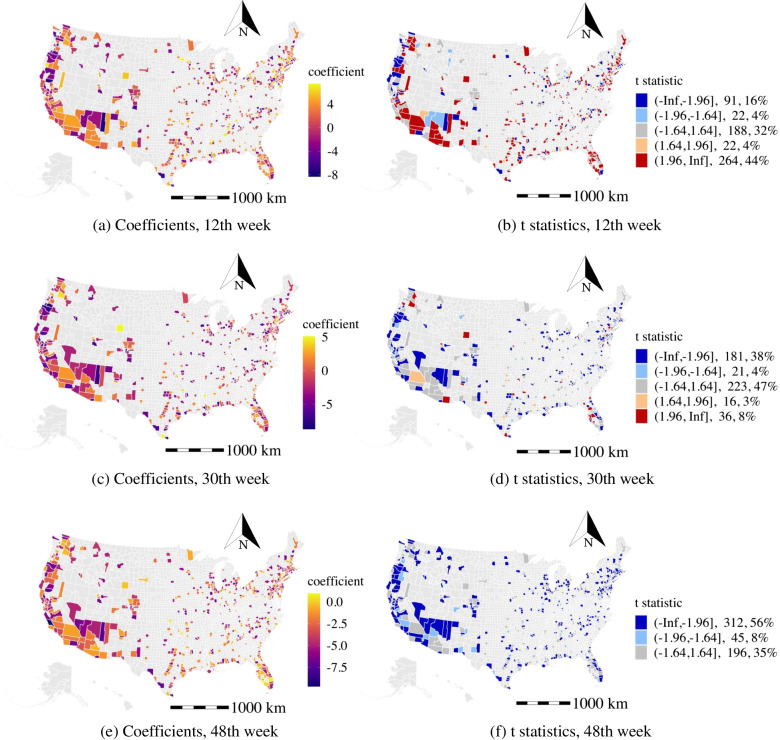
The distribution of the effects of the percentage of older population. Note: the range of Z-scores, the number of counties in the range, and the percentage of counties in the range are calculated in the t statistic legend (all the figures below follow the same approach)

In Fig. 5, we visualize the coefficient and t-stats distributions for the percentage of older population from three selected weeks (12th, 30th, 48th). The coefficients are observed to be positive, and the estimates are statistically significant in around 50% of the studied counties at the 12th week. However, the corresponding coefficients among 34% of these counties gradually shift to negative while the estimates remain statistically significant at the 48th week. One reason is that the older population is more vulnerable to the disease. This, along with the worse pandemic situation, indicates that the older population is increasingly cautious and adopting better preventative measures, which reduce their chances of being infected as the outbreak proceeds. In addition, among the studied counties that have insignificant effects at the 12th week, 59% of these counties have shifted to have negative coefficients and remain statistically significant at the 48th week. The coefficients of the high population density counties (mean population density is 357 population$/km^{2}$) are more likely to shift to negative in the later stage than the relatively low population density counties (mean population density is 288 population$/km^{2}$). That might be because the high population density counties provide a better guide for the older population in preventing the COVID-19. Finally, the coefficients are observed to be negative and statistically significant in around 64% of the studied counties at the 48th week.

#### Percentage of the Hispanic Latino population (LATP)

From the race perspective, the coefficients of the percentage of the Hispanic Latino population are positive, and the estimates are statistically significant in about 18% of the counties in the 12th week. later on, the percentage of Hispanic Latino population is statistically significant and positively related to the weekly average daily confirmed cases at over 90% of counties in the 30th week and 48th week.

#### Percentage of the Asian population (ASIP)

**Fig. 6:**
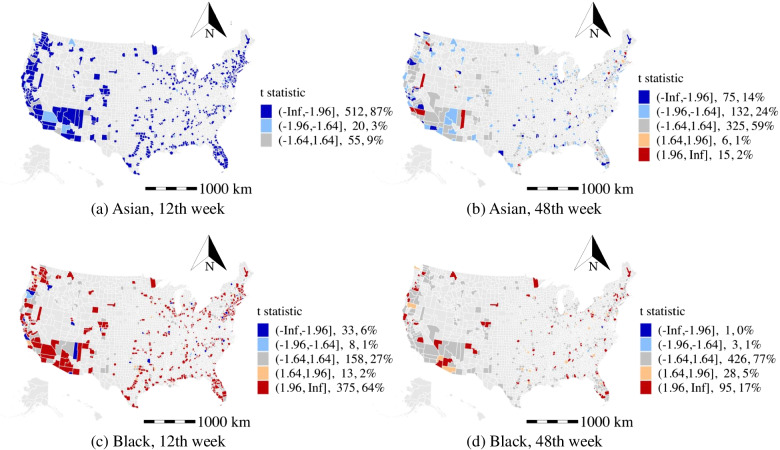
The t statistic distribution of race type

As shown by the t-stat in Fig. 6a and b, more than 40% of counties are observed to have negative coefficients of the percentage of the Asian population during the study period, which is more than the number of counties having positive coefficients, including the high population density cities and states (e.g., Californian, Seattle, and Florida). As reported by the studies [44, 45] that Asians have a higher infected rate by the COVID-19 due to the deficiency of Vitamin D and a higher incidence of coronary heart disease. However, the Asians might get warnings from their families and peers who experienced the earliest suffering of the COVID-19, which may help the Asian population to be more aware of the risk of the COVID-19 and may take better preventative actions against the disease in advance. It yields similar results when we apply the normalization of the race population across the studied counties.

#### Percentage of black or African American population (BLAP)

As shown in Fig. 6c, and d, the percentage of black or African American population is found to be a statistically significant factor in 70% of the counties in the 12th week, and the number of counties with significant effects drastically reduces to 20% in the 48th week. This finding is consistent with the previous survey [18], where they asserted that the black communities are more vulnerable due to the spread of the COVID-19 with the lower coverage rate of health insurance. The insights also indicate the black population has taken action to protect themselves, which reduces the chances of being infected in the late stage of the pandemic.

### The impacts of the socioeconomic variables

#### Citizen labor force unemployment rate (CLUE)

**Fig. 7:**
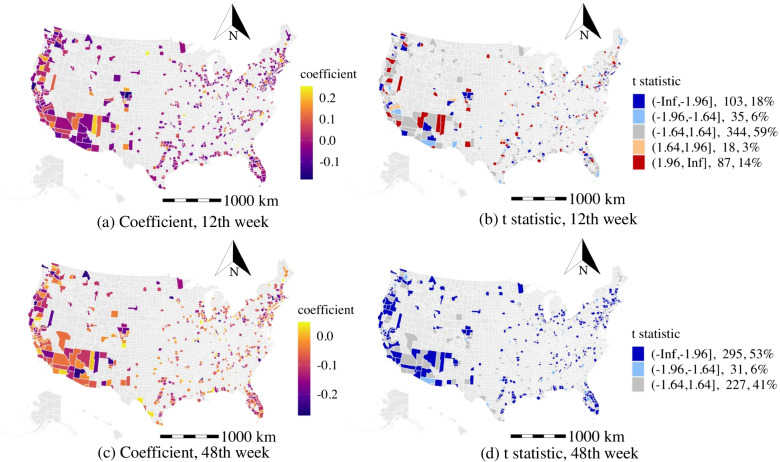
The distribution of the effects of citizen labor force unemployment rate

As for the effects of the socioeconomic variables, the estimates of the citizen labor force unemployment rate are statistically significant (t-stat > 1.64 or t-stat < -1.64) in around 50% of the counties during the study period (see Fig. 7). In the 12th week, the number of counties with a positive coefficient of the citizen labor force unemployment rate is more than the number of areas with negative effects (24% vs. 17% ). The unemployed population with unstable(unsafe) workplaces and irregular social activity might intensify the disease propagation as suggested by the previous finding [46]. More importantly, the high population density areas are more likely to have positive and statistically significant effects of the citizen labor force unemployment rate in the 12th week (e.g., counties in California, Washington, Arizona, Minnesota, and Florida). However, this situation has changed in the 48th week as shown in Fig. 7d. That may be related to the effectiveness of the shelter policy and the COVID subsidies, which mitigated the sufferings of the unemployed population in purchasing daily needs and helped reduce their daily activity levels. Similarly, the study [47] also indicates that occupation is the key factor affecting travel time change and infected rate.

#### Percentage of the population having at least bachelor’s degree (BDHP)

For the education effects, about 30% of areas have negative coefficients (t-stat < -1.64 ) of the percentage of the population having at least a bachelor’s degree, and about 10% of areas have positive coefficients (t-stat > 1.64) during the study period. The reason might be the highly educated population have a higher awareness of the risks of the COVID-19 and are more acceptable to the public suggestions for preventing the COVID-19.

#### Yearly mean household income (MHIC)

For the effects of the household income, the median elasticity indicates a 1% increase in the yearly mean household income results in a 0.83% increase in weekly average daily confirmed cases. The reason might be that commercial activity and business communication are more active in counties with high yearly mean household income.

### The impacts of the travel-related variables

#### Public road mileage (MILE)

As for the travel-related effects, the effects of the public road mileage are statistically significant and positive in all studied counties during the study period. It indicates that the availability of public transportation facilities is more likely to increase the number of weekly average daily confirmed cases. In particular, the median elasticity of the estimates suggests that a 1% increase in the public road mileage results in a 1.03% increase in the weekly average daily confirmed cases.

#### Mean commuting time (MTAT)

For the travel mode effects, the median elasticity shows that a 1% increase in the mean commuting time results in 0.22%(0.95%) increase in the weekly average daily confirmed cases. The effects of mean commuting time on the weekly average daily confirmed cases are heterogeneous among counties during the study period. For example, the mean commuting time is positively (t-stat > 1.64) related to weekly average daily confirmed cases for counties in California. However, the counties in Arizona have negative effects (t-stat < -1.64) of the mean commuting time on the weekly average daily confirmed cases. The underlying reason might be that the long mean commuting time in high population density areas intensifies disease propagation. Whereas the large-scale of urban structure with low population density effectively reduces the contact space among people.

#### Percentage of population taking public transit (PTAT)

**Fig. 8:**
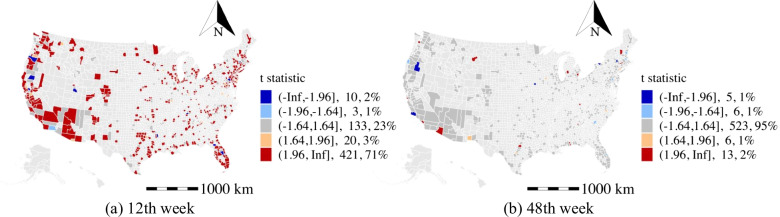
The t statistic distribution of percentage of population taking public transit

The t statistic estimations for the percentage of population taking public transit are summarized in Fig. 8. In particular, about 80% of counties have positive coefficients in the 12th week (see Fig. 8a).The long contact duration and close proximity among passengers in the transit system are likely the causes of this observation [22]. It also indicates that the public transit closure strategy may effectively curb the propagation of the COVID-19 in the early stage. However, the effects of the percentage of population taking public transit in most counties became less significant or insignificant in the 48th week. This highlights the temporally varying effects of the modeling determinants and suggests that the percentage of population taking public transit is no longer a determining factor in the later stage of the pandemic.

#### Percentage of population work at home (WFHP)

**Fig. 9:**
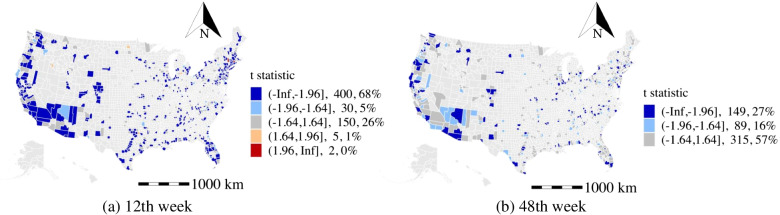
The t statistic distribution of percentage of population work at home

As shown in Fig. 9, there is a higher number of counties showing that the percentage of population work at home is negatively related to weekly average daily confirmed cases in the 12th week than in the 48th week. This finding is consistent with the estimated results of the percentage of population taking public transit and suggests that the work from home policy plays a more significant role in the early stage of the pandemic. Besides, the evidence of work from home policy applied in Australia [48] also verifies our insights.

### The impacts of the human activity variables

#### Change of grocery and pharmacy activity (GPPC)

**Fig. 10:**
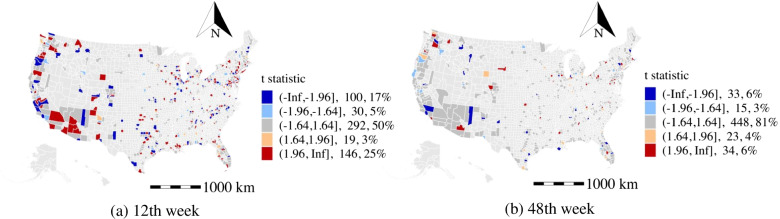
The t statistic distribution of change of grocery and pharmacy activity

The local daily activities directly respond to the work from home policy and travel restriction. Thus, the estimated coefficients of the human activity variables may serve as a measure of the effectiveness of disease prevention and mitigation strategies. In the M-GTWR model, the change of grocery and pharmacy activity is positively (t-stat>1.64) related to weekly average daily confirmed cases in about 30% of the counties in the 12th week of 2020 as shown in Fig. 10a. More importantly, the positive effects are found to be significant in rural counties or low-income areas in California (e.g., Tulare), Arizona (e.g., Yavapai), and New York (e.g., Long Island area). This is opposite to the estimated effects of the recreation and park activities (not present in the model due to the high correlation with the change of grocery and pharmacy activity) in the high population density areas. Although daily activities increase the disease propagation, people might have better personal prevention (e.g., social distance and mask-wearing) or reduce their daily activities in the high population density areas. Besides, the effects of the change of grocery and pharmacy activity on weekly average daily confirmed cases become less significant in most of the counties in the 48th week (see Fig. 10b).

#### Change of workplace activity (WOPC)

The change of workplace activity is either negatively (t-stat<-1.64) or insignificantly related to weekly average daily confirmed cases in most counties. That might be because the work from home policy conducted at the beginning stage of the pandemic reduces the work trips and workplace activities.

#### Change of transit stations activity (TSPC)

**Fig. 11:**
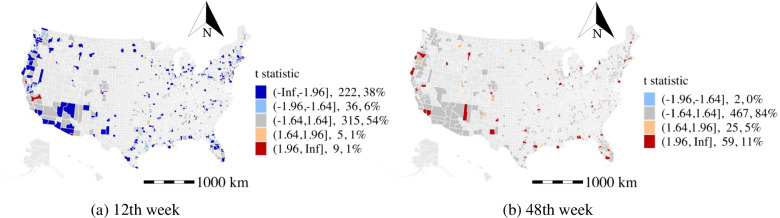
The t statistic distribution of change of transit stations activity

The effects of the change of transit stations activity are significant in areas with high population density (e.g., Fresno county in California) and low population density areas (e.g., Apache county in Arizona). These might be related to the transit usage policies that apply in different areas. And this discrepancy in terms of the change of transit stations activity highlights the importance of modeling spatial heterogeneity to more accurately understand the impacts of non-epidemiological factors. The distribution of the t stats of public transit is shown in Fig. 11). In the 12th week, the number of counties having negative coefficients of the change of transit stations activity on weekly average daily confirmed cases is more than the number of counties having positive coefficients. However, this situation has changed in the 48th week. This might be related to the public transit usage restrictions in the early period of the pandemic and the reopening strategy at the later stage. Therefore, the analysis shows that the effects of the influencing factors are spatiotemporal heterogeneous.

In conclusion, based on our analysis, the coefficients of the population density and public road mileage yield persistently positive and statistically significant in studied counties during the studied period. This clarifies that population density and public infrastructure facilities are the primary factors that intensify the number of cases during the pandemic. On the other hand, the impacts of the several social-demographic variables (e.g., the percentage of black population, yearly mean household income, and percentage of the population taking public transit) are observed to become less significant or even insignificant in the later weeks (e.g., the 48th week). This suggests that static variables may have greater impacts on the disease dynamics in the early stage of the pandemic than in the later stage. Nevertheless, the human daily activity variables (e.g., change in workplace activity and change in transit stations activity) are sensitive to the disease prevention policies, and their impacts remain statistically significant during the entire course of the COVID-19.

## Discussion

In this study, we developed an M-GTWR model to investigate the effects of non-epidemiological factors on disease propagation. Specifically, we show that the proposed M-GTWR model is superior to the state-of-the-art benchmarks in capturing the spatiotemporal heterogeneity of disease dynamics during the COVID-19 outbreak. Our results find that the older, the black, and the Latino are more vulnerable to the COVID-19 than other population groups. The reason may be attributed to either physical weakness or low-risk awareness. The highly educated population is more likely to comply with the restrictions during the COVID-19 outbreak. For the commuting time, its median elasticity shows that a 1% increase in the commuting time to work results in a 0.22% increase in the weekly average daily confirmed cases. Finally, the change in human activity patterns also presents a mixed impact on disease dynamics. In particular, the scale of the impacts is found to be closely related to the activity intensity and activity types. The grocery and pharmacy activity is found to be significant in low population density areas. And activities associated with public transit usage lead to a positive impact on the weekly average daily confirmed cases. This indicates the major role played by the public transit during COVID-19 and implies the need to restrict public transit usage, especially in high-transit demand areas. These insights address the spatiotemporal effects of the non-epidemiological factors on the COVID-19 propagation.

Several implications for the high population density areas (e.g., New York City, counties in California, Washington, Arizona, Virginia, Minnesota, and Florida):

1. The intensity of recreation activity is found to be a primary activity factor that facilitates the spread of the COVID-19. Besides, limiting access to public transit and public office is observed to be effective during the pandemic as suggested in Fig. 11.
2. Among the counties with a high population density, the percentage of the unemployed population (see Fig. 7) and population with a low education level are the two primary factors associated with a higher number of weekly average daily confirmed cases.
3. High population density areas may spend more resources on the older population to reduce the exposure rate, especially in public areas, as suggested in the aforementioned analysis of the older population.
4. High population density areas with a high percentage of black population may consider spending more efforts in alerting the black communities on the risk of the COVID-19 and enforcing the adoption of personal protective equipment such as face masks.

Several implications in our study that are important for the low population density counties (e.g., counties in Arizona and counties in Massachusetts):

1. The work from home policy and public transit restriction may be ineffective. Instead, the low population density areas may focus on providing specific strategies to regulate the daily activities of the unemployed populations as suggested in Fig. 7a and b.
2. The low population density counties should advise the older population to avoid riding public transit and visiting public recreation areas.
3. The racial disparities in the infections of the COVID-19 are especially significant in low population density (e.g., counties in New Mexico, Arizona, and Massachusetts). The black community suffers more than other races in most of the low population density counties (see Fig. 6c and d). Besides, counties in Utah may benefit from improving the COVID-19 prevention among Asian communities (see Fig. 6a and b).

The study explores the spatiotemporal effects of non-epidemiological factors on the COVID-19 propagation and addresses the heterogeneous effects of demographic characteristics and daily activity on disease propagation. However, there are some limitations in the study. First, the efficiency of the intervention strategies (e.g., wearing face masks, maintaining social distance, and handwashing) for mitigating the spreading of COVID-19 lack of exploration due to the limited data source. More importantly, since these strategies are at a great cost to the economy, the optimal control strategies to balance public health and freedom of movement, the economy, and society deserve further investigation. Second, although we estimated the effects of the several types of activities, we do not differentiate the risk level of detailed activities due to the data limitation (e.g., we estimated the effects of the recreation activities, but the exposure risk of the bar and book store might be different). The understanding of the exposure risk of detailed activities provides directional suggestions for the policy-makers in conducting control strategies for COVID-19 prevention. Third, the findings rely on the analysis of the aggregated county-level dataset. However, the lack of exploration of the microscopic behavior-related analysis would increase the uncertainty of underlying reasons. Thus, future studies should be more tailored to the demographics and socioeconomic of the particular location and groups. Besides, we used the sampling of US counties to construct the model. The applicability of insights remains to be tested for the rest of counties in the US and other countries. The model parameters can also be adjusted using the data from other locations.

## Conclusion

By establishing a quantitative framework for identifying influencing factors of COVID-19 dynamics in the US, the study first concludes that the proposed M-GTWR achieves a substantial improvement over other benchmark methods in addressing the spatiotemporal nonstationarity issues in the disease dynamic data. Then, we obtain several key results from the study. High population density and the availability of public infrastructures will facilitate the spread of the disease. A 1% increase in population density and public road mileage leads to 0.63% and 1.03% more daily cases on average, respectively. Besides, the effects of socio-demographic attributes and the travel-related attributes differ significantly over time and the underlying location. Moreover, the effectiveness of limiting human contact through reduced human activity levels is found to vary significantly over space and time. The grocery and pharmacy activity is positively related to daily cases in about 30% of studied counties in the 12th week of 2020. This number decreases to 10% in the 48th week of 2020. This reveals that the general preventative non-pharmaceutical measures, such as work from home policy and travel restrictions, are unlikely to be universally effective over all subareas of a country. The insights derived in this study will provide important guidance for efficient resource allocation strategies (e.g., the distribution of medical resources) and non-pharmaceutical interventions for future disease mitigations and interventions.

## Data Availability

The datasets generated and/or analysed during the current study are available in the the Center for Systems Science and Engineering at the Johns Hopkins University [1], the Google Mobility Report [31], US Census Bureau’s MAF/TIGER Geodatabases [32], the US Department of Transportation and Bureau [36], and and the 2016 American Community Survey [33].

## Abbreviations

COVID-19: Coronavirus disease 2019
M-GTWR: mobility-augmented geographically and temporally weighted regression
M-GWR: mobility-augmented weight regression
SARS-CoV-2: severe acute respiratory syndrome coronavirus 2
GTWR: geographically and temporally weighted regression
GWR: geographically weighted regression
TSPC: change of transit stations activity
WOPC: change of workplace activity
GPPC: change of grocery and pharmacy activity
WFHP: percentage of population work at home
PTAT: percentage of population taking public transit
MTAT: mean commuting time
MILE: public road mileage
MHIC: yearly mean household income
BDHP: percentage of the population having at least bachelor’s degree
CLUE: citizen labor force unemployment rate
ASIP: percentage of Asian population
BLAP: percentage of black or African American population
LATP: percentage of Hispanic Latino population
OLDP: percentage of older population
POPD: population density
OLS: ordinary least squares
AIC: Akaike Information Criterion
AICc: corrected Akaike Information Criterion
CV: cross-validation
BP: Breusch-Pagan
VIF: variance inflation factor
ANOVA: analysis of variance
